# The Dishevelled, EGL-10 and Pleckstrin (DEP) Domain-Containing Protein DEPDC7 Binds to CARMA2 and CARMA3 Proteins, and Regulates NF-κB Activation

**DOI:** 10.1371/journal.pone.0116062

**Published:** 2014-12-26

**Authors:** Egildo Luca D′ Andrea, Angela Ferravante, Ivan Scudiero, Tiziana Zotti, Carla Reale, Maddalena Pizzulo, Luigi Regenburgh De La Motte, Chiara De Maio, Pellegrino Mazzone, Gianluca Telesio, Pasquale Vito, Romania Stilo

**Affiliations:** 1 Dipartimento di Scienze e Tecnologie, Università degli Studi del Sannio, Via Port′Arsa 11, 82100 Benevento, Italy; 2 Biogem Consortium, Via Camporeale, 83031 Ariano Irpino (AV), Italy; 3 SannioTech Consortium, Strada Statale Appia, Benevento, Italy; IISER-TVM, India

## Abstract

The molecular complexes containing BCL10, MALT1 and CARMA proteins (CBM complex) have been recently identified as a key component in the signal transduction pathways that regulate activation of Nuclear Factor kappaB (NF-κB) transcription factor. Herein we identified the DEP domain-containing protein DEPDC7 as cellular binding partners of CARMA2 and CARMA3 proteins. DEPDC7 displays a cytosolic distribution and its expression induces NF-κB activation. Conversely, shRNA-mediated abrogation of DEPDC7 results in impaired NF-κB activation following G protein-coupled receptors stimulation, or stimuli that require CARMA2 and CARMA3, but not CARMA1. Thus, this study identifies DEPDC7 as a CARMA interacting molecule, and provides evidence that DEPDC7 may be required to specifically convey on the CBM complex signals coming from activated G protein-coupled receptors.

## Introduction

The NF-κB signaling pathway is a major regulator of normal immune and inflammatory responses, cell proliferation, differentiation, apoptosis and oncogenesis [1].

Previous studies have demonstrated that signal-dependent formation of the CARMA-BCL10-MALT1 complex (known as the CBM complex) recruits downstream signaling components that modulate activation of NF-κB transcription factor [2]–[4]. Key component of the CBM complex are the three CARMA proteins, CARMA1, 2 and 3, which constitute a family of proteins conserved across many species and are characterized by the presence of different functional domains shared by all members of the family [2], [3]. Structurally, all CARMA proteins contain a CARD domain located at their amino-terminus, followed, in order, by a coiled coil domain, an SH3 domain, and a guanylate kinase-like domain (MAGUK) located at the carboxy-terminus of the protein [2], [3]. The expression pattern of the three CARMA proteins is however different for the individual molecules. Indeed, CARMA1 is mostly expressed in lymphoid tissues [5], while CARMA2 and CARMA3 are non-overlappingly expressed in a variety of different tissues [6]–[8]. This characteristic expression pattern of CARMA protein has corroborated the speculation that they may perform similar functions in different tissues.

Genetic and biochemical studies have identified CARMA1 as crucial component of the CBM complex that links antigen receptors on B and T lymphocytes to activation of NF-κB. In mice, genetic inactivation of CARMA1 results in a complete block in T and B cell immunity, being lymphocytes from CARMA1-deficient mice severely impaired in antigen receptor-mediated proliferation and cytokine production due to a selective defect in JNK and NF-κB activation [9]–[11].

Similarly, a CBM complex that comprises CARMA3, BCL10 and MALT1, plays an essential role in activation of NF-κB in cells outside of the immune system [12]. In fact, independent studies have established that CARMA3 is implicated in the signal transduction pathways elicited by G protein-coupled receptors (GPCRs), a large family of cell surface receptors that regulate cell migration, differentiation, proliferation and survival [13]–[15]. Recent data also show that the less characterized members of the CARMA family of proteins, CARMA2 and its splice variants, regulate activation of NF-κB through formation of an analogous CBM-complex [8].

The Dishevelled, EGL-10 and pleckstrin (DEP) domain is a globular protein domain that, despite having diverse mechanisms of action, generally assists translocation of the cognate protein to the plasma membrane [16]. Some DEP domain-containing proteins contain both a DEP domain and a Regulator of G protein signalling (RGS) domain, which confers GTPase-activating protein (GAP) activity. In fact, similarly to CARMA3, DEP-containing protein are mostly implicated in the signal transduction pathways starting from GPCRs [16].

Here, we demonstrate that the DEP domain containing protein DEPDC7 associates to CARMA2 and CARMA3. The functional significance of this interaction is highlighted by the evidence here shown that shRNA-mediated DEPDC7 depletion results in a marked reduction of NF-κB activation following stimuli that require correct assembly and functioning of the CBM complex.

## Materials and Methods

### Reagents

Sources of antibodies and reagents were the following: anti-FLAG, anti-HA, anti-βactin, anti atubulin Sigma; anti-Net1, anti-myc, Santa Cruz Biotechnology; anti-CARMA3 has been generated in our laboratory and has been described elsewhere [17]. Recombinant tumor necrosis factor α (TNFα) was from Milteny; interleukin-1β (IL-1β lysophosphatidic acid (LPA), phorbol myristic acid (PMA) and ionomycin were obtained from Sigma.

### Two-hybrid Screenin

The two-hybrid screening was performed using the Matchmaker system (Clontech) as previously described [18]. Briefly, yeast strain AH109 GAL4^−/−^ was first transformed with pGBKT7 plasmids carrying a cDNA bait fused with DBD of GAL4 using the lithium acetate/PEG 3000 procedure. Transformant colonies were selected on synthetic dropout plates lacking tryptophan. Expression of bait fusion proteins was assessed by immunoblot analysis. For library screening, yeast AH109 expressing GAL4DBD-CARMA2*sh* [8] was transformed with a human cDNA library cloned in pACT2 vector (Clontech) in fusion with GAL4TAD. 2×10^6^ clones were screened for interaction with GAL4DBD-CARMA2*sh* using selective growth on minimal medium lacking nutrients whose biosynthesis is mediated by genes under control of GAL4 transcriptional activity.

### Real-time PCR

Real-time PCR reactions were performed in triplicate by using the SYBR Green PCR Master Mix (Qiagen) in a 7900HT sequence-detection system (Applied Biosystems). The relative transcription level was calculated by using the ΔΔCt method.

### Generation of DEPDC7 polyclonal antibody

A cDNA fragment encoding the polypeptide Thr3-Ser164 of DEPDC7 was cloned into pET-28a (Novagen) to create an N-terminal Histidine-tagged fusion protein. The His fusion protein was purified by affinity chromatography on Nichel-Sepharose beads as previously described [19]. The purified polypeptide was used for the immunization of two rabbits to obtain polyclonal antibodies (InCura srl, Italy).

### Immunoblot analysis and coprecipitation

Cell lysates were made in lysis buffer (150 mM NaCl, 20 mM Hepes, pH 7.4, 1% Triton X-100, 10% glycerol and a mixture of protease inhibitors). Proteins were separated by SDS-PAGE, transferred onto nitrocellulose membrane and incubated with primary antibodies followed by horseradish peroxidase conjugated secondary antibodies (Amersham Biosciences). Blots were developed using the ECL system (Amersham Biosciences). For coimmunoprecipitation experiments, cells were lysed in lysis buffer and immunocomplexes were bound to protein A/G (Roche), resolved by SDS-PAGE and analyzed by immunoblot assay.

### Cell culture and transfection

HEK-293 cells were obtained from ATCC and maintained in Dulbecco's modified Eagle's medium (DMEM) supplemented with 10% FBS. DNA plasmids were transfected into cultured cells by calcium-phosphate methods or using Lipofectamine2000 (Invitrogen) according to the manufacturer's protocol. Retroviral infections were performed as previously described [20].

### Luciferase Assay

To assess for NF-κB activation, HEK-293 cells were plated in standard 6-well plates with with 0.2 µg of pNF-κB-luc (Clontech), 0.1 µg of pRSV-βGal (Addgene) plus each expression plasmid. When necessary, the total amount of transfected plasmidic DNA (2 µg) was kept constant by adding empty vector. 16 hrs after transfection, NF-κB activation was assessed by measuring luciferase activity with the Luciferase Assay System (Promega) normalized on β-galactosidase activity.

### Immunofluorescence

1×10^4^ HEK-293 cells were seeded in chamber slides. 24 hrs later, cells were fixed in 3% paraformaldehyde for 10 minutes at room temperature and then permeabilized in 0,2% Triton X-100 for 5 minutes. Cells were incubated for 1 hr with primary antibody, followed by three washes with PBS and a second incubation with secondary antibodies FITC-conjugated for 1hr. After three washes with PBS, nuclei were stained with DAPI for 5 minutes. Then, chamber slides were dried and analyzed with fluorescence microscopy. All steps were done at room temperature.

### Wound healing assay

Cells were seeded onto Petri dishes and cultured at 37°C for 24 hrs. The cell monolayer was then wounded with a plastic tip, and examined using a light transmission microscope connected to a camera to capture the images. Wound width was measured with the ImageJ software.

## Results

### Two-hybrid Screening

To identify novel interactors of CARMA proteins, we carried out two-hybrid screenings using as a bait CARMA2*short* (CARMA2*sh*) fused to the DNA-binding domain of GAL4. Plasmid libraries of fusion between the transcription activation domain of GAL4 and cDNAs from human tissues were screened for interaction with GAL4-CARMA2*sh* in the yeast reporter strain AH109. Several clones were isolated that activated the β-galactosidase reporter gene. Sequence analysis revealed that several isolated plasmids, identified recurrently in multiple independent copies, encoded for the full length sequence of DEPDC7. Further assays performed with one of these library plasmids confirmed the interaction of DEPDC7 with CARMA2*sh* in the reporter yeast strain (Table 1).

**Table 1:** Interaction of DEPDC7 with CARMA2*sh* in the yeast two-hybrid assay.

Protein fused to GAL4 domain		Yeast growth on selective media
DNA-binding	Activating	
-	**DEPDC7**	-
**Vector**	**DEPDC7**	-
**FADD**	**DEPDC7**	-
**CARMA2** ***sh***	**DEPDC7**	**+**

Yeast AH109 was transformed with DEPDC7 fused to the GAL4-TAD, together with the indicated cDNAs fused to GAL4-DBD. The cDNA encoding for FADD served as a negative control. The interactions were examined by yeast growth on selective media; assays were done for five to ten independent transformants. Yeast colonies were scored as positive when growth developed within 24–36 hrs; a negative was scored when growth failed to develop within 1 week.

**Figure 1:**
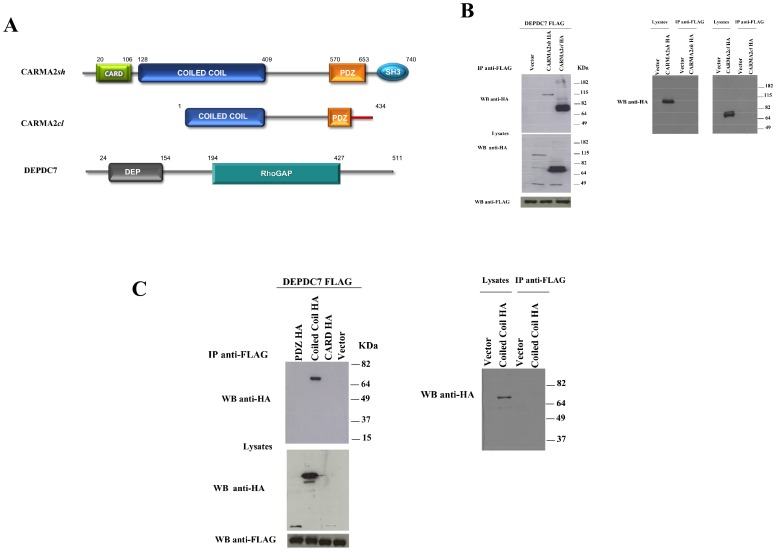
CARMA2sh and CARMA2cl isoforms interact with DEPDC7. A) Schematic representation of CARMA2*sh*, CARMA2*cl* and DEPDC7. B-C) HEK-293 cells were transiently cotransfected with a FLAG-tagged version of DEPDC7 along with the HA-tagged version of the indicated constructs. 24 hrs later, cell lysates were prepared and immunoprecipitated with anti-FLAG mAb. Immunocomplexes were separated by SDS-PAGE and transferred onto membranes, subsequently probed with anti-HA antibody. Right panels show controls for the specificity of immunoprecipitation.

To test for a physical association between CARMA2*sh* and DEPDC7 in mammalian cells, HEK-293 cells were cotransfected with plasmids expressing FLAG-tagged DEPDC7 together with a vector encoding for HA-tagged CARMA2*sh* or CARMA2*cardless* (CARMA2*cl*) (Fig. 1A). Cell lysates were immunoprecipitated with anti-FLAG-coated beads, and the presence of coprecipitating CARMA2 isoforms was assessed by immunoblot experiments with anti-HA antibody. The results shown in Fig. 1B indicate that both CARMA2 isoforms coprecipitate with DEPDC7, suggesting that the region of interaction of CARMA2 with DEPDC7 resides in the coiled coil/linker region of the protein (see Fig. 1A). In fact, experiments performed with deletion mutants of CARMA2 indicate that the region 123–550 of the protein, encompassing the coiled coil and the linker region, associates with DEPDC7, while the CARD domain (1–123) and the PDZ domain (550–690) do not (Fig. 1C).

**Figure 2:**
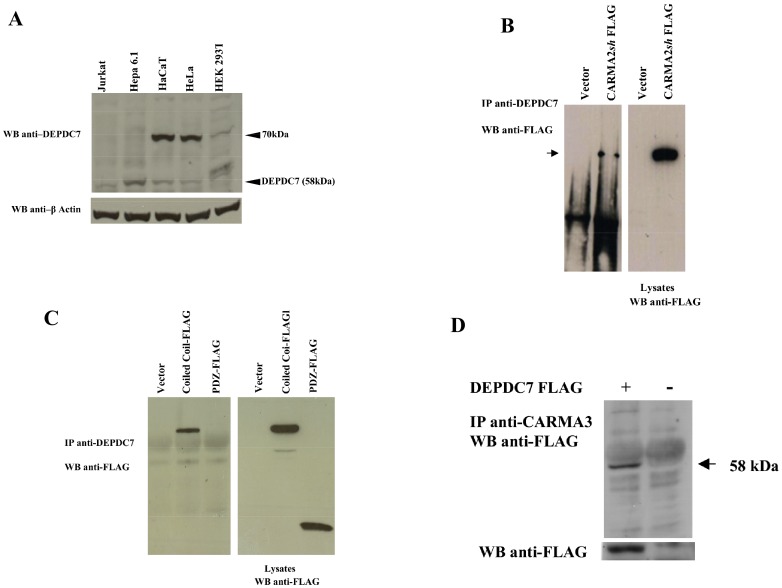
CARMA proteins associates with endogenous DEPDC7. A) Total cell lysates (30 µg) prepared from the indicated cell lines were separated by SDS-PAGE and transferred onto membranes subsequently probed with the affinity purified anti-DEPDC7 antisera. B-D) HEK-293 cells were transiently cotransfected with FLAG-tagged versions of the indicated constructs. 24 hrs later, cell lysates were immunoprecipitated with anti-DEPDC7 (B, C) or with anti-CARMA3 (D) antisera and analyzed for coprecipitating proteins by immunoblot assay.

In order to characterize endogenous DEPDC7, we generated a rabbit antiserum against DEPDC7 using as antigen a recombinant polypeptide produced in E. *coli* encompassing the amino acids Thr3-Ser164 of DEPDC7. This affinity purified antiserum recognizes a band with the expected molecular mass of 58 kD in proteic lysates from different cell lines (Fig. 2A). In addition to the band at 58 kD, lysates from some cell lines, including HeLa and HaCaT cells, also show a specific band at 70 kD, suggesting that these cell lines may express different isoforms of DEPDC7 (Fig. 2A). Coprecipitation experiments conducted using the anti DEPDC7 antiserum indicate that endogenous DEPDC7 coprecipitates with transfected CARMA2*sh* (Fig. 2B). Moreover, experiments with deletion mutants confirmed association of the coiled coil/linker region of CARMA2 with endogenous DEPDC7 (Fig. 2C). Finally, in addition to CARMA2*sh*, DEPDC7 also coprecipitates with CARMA3 (Fig. 2D).

**Figure 3:**
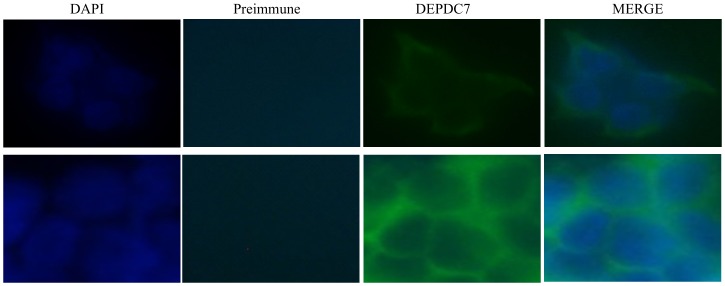
Cytosolic subcellular localization of DEPDC7. HEK-293 cells were stained with anti-DEPDC7 antisera followed by FITC-conjugated anti-rabbit IgG; nuclei were stained with DAPI. Two different fields for each sample are shown.

A set of fluorescence microscopy experiments, shown in Fig. 3, indicated that endogenous DEPDC7 displays a cytosolic distribution, somehow more concentrated in the vicinity of the cell membrane.

**Figure 4:**
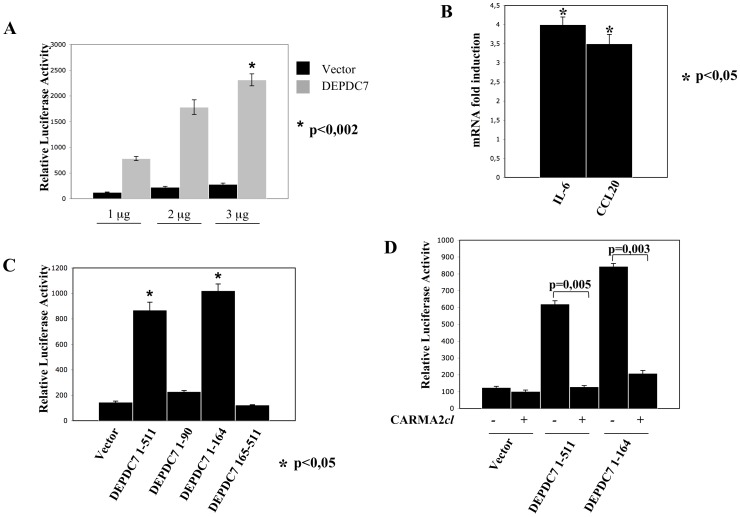
DEPDC7 activates NF-κB. A) HEK-293 cells were co-transfected with pNF-κB-luciferase plasmid, a β-galactosidase reporter vector, and the indicated amount of an expression vector encoding for the DEPDC7. 24 hrs after transfection, cell lysates were prepared, and luciferase activity was measured. Data shown represent relative luciferase activity normalized against β-galactosidase activity and are representative of at least 10 independent experiments performed in triplicate. B) Real-time PCR analysis of IL-6 and CCL20 mRNAs in cells transfected with DEPDC7. Data shown indicates normalized fold induction over mock transfectant. Statistical analysis was performed by Student's t test; a p value of <0.05 was considered significant. C-D) HEK-293 cells were co-transfected with pNF-κB-luciferase plasmid, a β-galactosidase reporter vector and expression vectors encoding for the indicated polypeptides. 24 hrs after transfection, cell lysates were prepared and luciferase activity was measured. Data shown is representative of at least 10 independent experiments performed in triplicate. Statistical analysis was performed by Student's t test; a p value of <0.05 was considered significant.

Because both CARMA2 and CARMA3 proteins are implicated in the NF-κB signaling pathways, we first investigated for an involvement of DEPDC7 in this pathway, by using a luciferase-based reporter assay. When DEPDC7 was expressed in HEK-293 cells in a dose-dependent manner, NF-κB activity was induced at least 8–10 fold compared with the empty vector (Fig. 4A). Induction of the transcriptional activity of NF-κB by DEPDC7 was confirmed by monitoring the expression level of two well known NF-κB target, namely IL-6 and CCL20 [21]–[23], which are both up-regulated upon DEPDC7 expression (Fig. 4B). Experiments performed using deletion mutants of DEPDC7 established that induction of NF-κB by DEPDC7 requires a functional DEP domain of the protein (Fig. 4C). Finally, DEPDC7-induced NF-κB activation is suppressed by CARMA2*cl*, a naturally occurring dominant negative form of CARMA2 [8] (Fig. 4D). From these experiments, we concluded that DEPDC7 acts as a positive regulator of NF-κB transcriptional activity, and for this it requires a functional CARMA protein.

**Figure 5:**
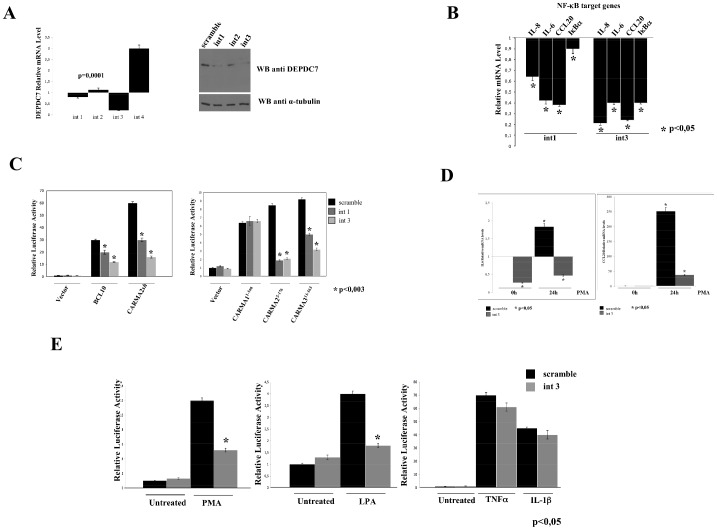
DEPDC7 depletion reduces NF-κB activation. A) HEK-293 cells were infected with retroviral vectors encoding for four different shRNAs targeting human DEPDC7 or a control shRNA (scramble). After selection, DEPDC7 mRNA levels normalized to GAPDH were quantified by real-time PCR. *Right panel*: protein lysates extracted from cells retrovirally expressing the indicated shRNA targeting DEPDC7 were analyzed for DEPDC7 expression by immunoblot assay B) Real-time PCR analysis of NF-κB target genes mRNAs in cells infected with retroviral vectors encoding shRNAs targeting human DEPDC7 or a control shRNA. Data shown indicates normalized expression level over scramble infected cells. C) HEK-293 cells depleted of DEPDC7 were transfected with pNF-κB-luciferase reporter plasmid and the indicated expression constructs. Data shown represents the relative luciferase activity normalized against β-galactosidase activity and is representative of at least 10 independent experiments performed in triplicate. Statistical analysis was performed by Student's t test; a p value of <0.05 was considered significant. D-E) Real-time PCR analysis of IL-6 and CCL20 mRNAs and NF-κB-driven luciferase activity in HEK-293 cells depleted of DEPDC7 following stimulation with PMA (40ng/ml), LPA (10 µM), TNFα (10 ng/ml) and IL-1β (20 ng/ml). Statistical analysis was performed by Student's t test; a p value of <0.05 was considered significant.

We next investigated the effect of short hairpin RNAs (shRNA)-mediated knockdown of DEPDC7 on the activation of NF-κB. For this, we used a retroviral expression system encoding shRNAs designed to target human DEPDC7 for degradation by the RNAi pathway. As shown in Fig. 5A, one shRNA targeting DEPDC7, designed int 3, produces a reduction of about 70–80% of the expression of DEPDC7 mRNA, whereas the shRNA int 1 results in a 10–20% reduction of DEPDC7 mRNA. An immunoblot assay confirmed that the observed reduction of DEPDC7 mRNA corresponds to a reduction of protein expression (Fig. 5A, right panel).

In HEK-293 cells, DEPDC7 silencing reduced the basal expression level of several NF-κB target genes, including IL-6, IL-8, CCL20 and I-κBα (Fig. 5B). Moreover, depletion of DEPDC7 dramatically reduced NF-κB activation elicited by several stimuli that require the CBM complex, such as expression of BCL10, CARMA2*sh*, and the CARD domains of CARMA2 and CARMA3, but not CARMA1 (Fig. 5C). More importantly, DEPDC7 abrogation results in impaired NF-κB activation following cellular stimulation with lysophosphatidic acid (LPA) and phorbol myristic acid (PMA) (Fig. 5D-E), two stimuli that require CARMA3 to activate NF-κB [13]–[15]. On the other hand, DEPDC7 depletion had no effect on NF-κB activation elicited by TNFα and IL-1β stimulation (Fig. 5E).

**Figure 6:**
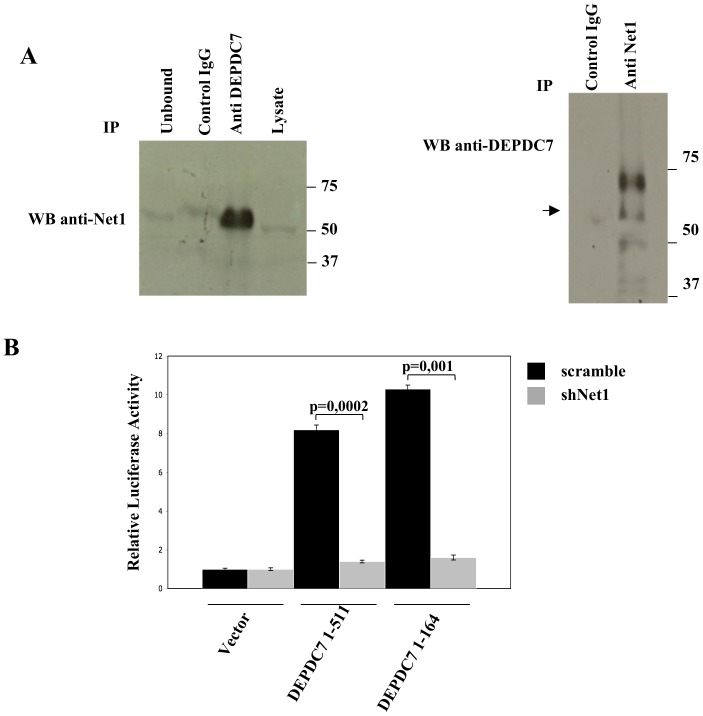
Net1 depletion reduces DEPDC7-mediated NF-κB activation. A) Cell lysates from HEK-293 cells were immunoprecipitated (IP) with the indicated antibodies and analyzed by immunoblotting. Anti-myc antisera (Santacruz) served as a control IgG. B) HEK-293 cells were infected with retroviral vectors encoding for shRNAs targeting Net1 or a control sequence (scramble). After selection, cells were transiently co-transfected with an expression vector encoding the indicated polypeptides, together with NF-κB-luciferase and β-galactosidase reporter vectors. 24 hrs after transfection, cell lysates were prepared, and luciferase activity was measured. Data shown represent relative luciferase activity normalized against β-galactosidase activity and are representative of at least 10 independent experiments performed in triplicate. shRNAs targeting Net1 have been described in [24]. Statistical analysis was performed by Student's t test; a p value of <0.05 was considered significant.

It is already known that the activity of the CBM complex is also regulated by Net1, a Rho guanine nucleotide exchange factor that binds to CARMA proteins and cooperates in activating NF-κB [24]. As DEPDC7 contains a Rho-GAP domain, we examined whether there is a functional relationship between Net1 and DEPDC7. Indeed, Net1 and DEPDC7 endogenously coprecipitate from HEK-293 cell lysates (Fig. 6A). More importantly, shRNA-mediated depletion of Net1 completely abrogates the NF-κB-inducing activity of DEPDC7 (Fig. 6B).

## Discussion

The three CARMA proteins have recently attracted considerable attention because they are involved in signal transduction pathways leading to activation of the transcription factor NF-κB from different membrane receptors [3]. To perform this function, each of the CARMA proteins forms a complex that contains BCL10 and MALT1 [2], [3]. While CARMA1 is involved in the signal transduction pathways arising following antigen receptor stimulation in lymphocytes, and is in fact exquisitely expressed in immune cells, CARMA3 shows a wider distribution, participating in the signal transduction pathway connecting GPCRs stimulation to NF-κB activation [13]–[15]. However, while it is fairly well known the mechanism by which the complex CBM works in lymphocytes [2], [3], is not clear as the CBM complex is recruited and activated following GPCRs stimulation. The data presented in this paper shed some light on this aspect. In fact, proteins containing a DEP domain have long been known to regulate recruitment of signal transducers to the receptor complex following GPCRs stimulation. Thus, in this context, DEPDC7 could be the linker molecule that connects activated GPCRs to the CBM complex. On the other hand, the evidence that the activity of DEPDC7 is functionally related to Net1, a Rho guanine nucleotide exchange factor, further supports this possibility.

At the moment there is no information in the literature on the biological function of DEPDC7. Therefore, our data showing that DEPDC7 positively regulates activation of NF-κB is the first information ever on this protein.

**Figure 7:**
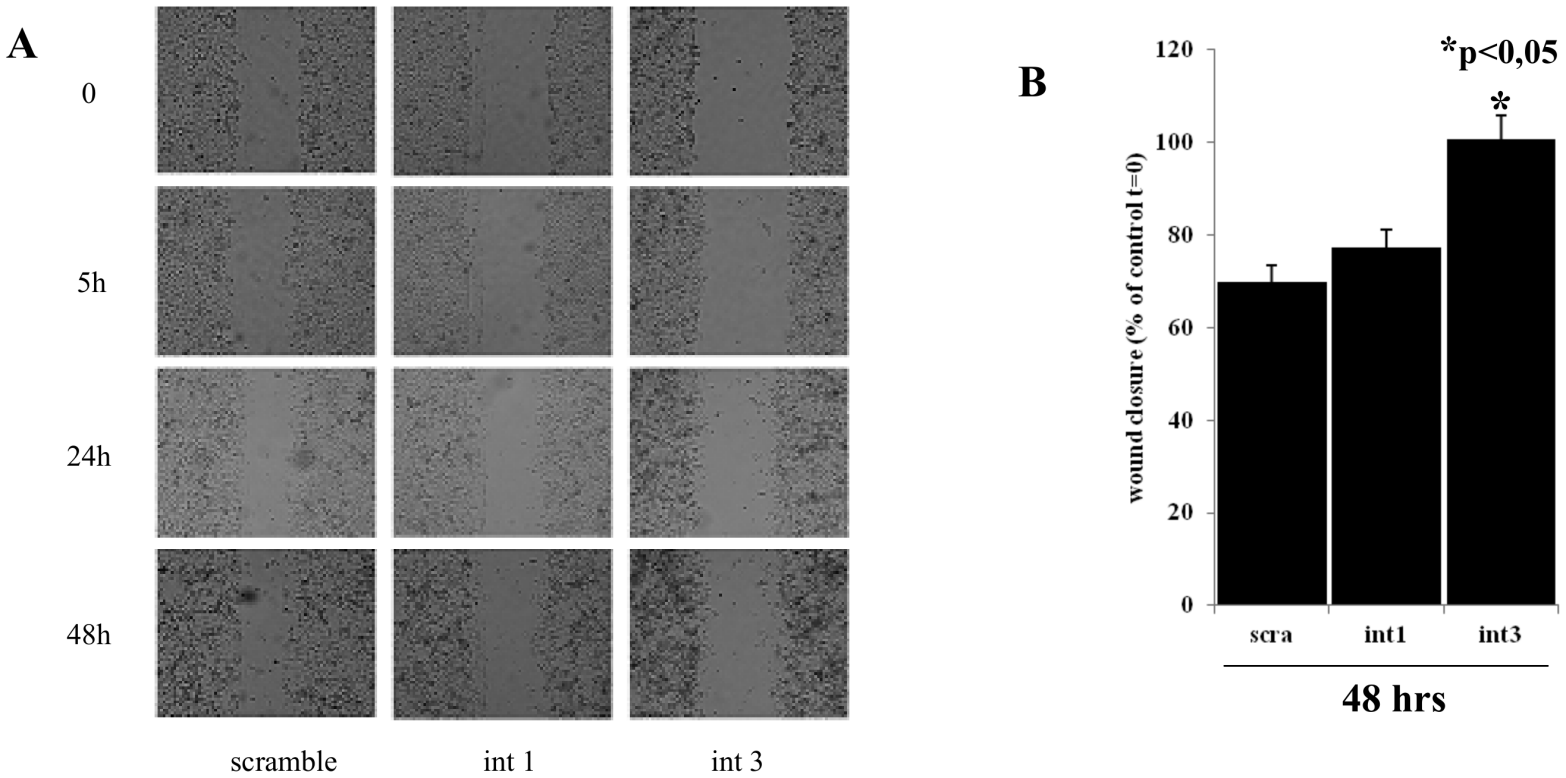
Cells lacking DEPDC7 show a reduced proliferation/migration capacity. A) HEK-293 cells depleted of DEPDC7 and control cells were cultured for 24 hrs. The cell monolayer was then wounded with a plastic tip, and examined at the indicated time points with a light transmission microscope. Data shown is representative of four independent experiments. B) Quantification of wound closure. Graphic represents the wound width as the mean s.e.m. of the % of the closure of original wound in triplicate plates after 48 hrs. Wound width was measured with the ImageJ software. Similar results were obtained in three experiments. Student's t-test was used to evaluate statistically significant differences between the values.

Abrogation of DEPDC7 expression mediated by specific shRNAs results in downregulation of the target genes transcribed by NF-κB that we monitored (Fig. 5). To test whether this effect is biologically relevant, we also conducted a wound healing test on cells interfered for DEPDC7 (Fig. 7). Although indirectly, this experiment demonstrates that cells lacking of DEPDC7 show a reduced proliferation/migration capacity, which is consistent with a reduced NF-κB activity.

Our biochemical and functional data indicates that DEPDC7 induces activation of NF-κB through the CBM complex. Furthermore, another evidence for a functional implication of DEPDC7 with the CBM complex derives from the fact that DEPDC7-mediated induction of NF-κB is inhibited by the A20 deubiquitinase (data not shown), as it happens for the CBM complex [25].

Finally, it is striking the evidence here provided that DEPDC7 seems to be required for the proper functioning of the CBM complex containing CARMA2 and CARMA3 but not CARMA1. This, however, is in agreement with previous findings showing that although the CARMA3/BCL10/MALT1 signalosome shares features with the CARMA1-containing signalosome, there are significant differences in how the signalosomes communicate with their cognate receptors [15]. This reinforces even more the possibility that DEPDC7 may be required to specifically convey on the CBM signals coming from activated GPCRs.

In conclusion, the work presented here certainly adds more details to our understanding of the molecular mechanisms that regulate the signal transduction pathways mediated by the CBM complex and the subsequent activation of the transcription factor NF-κB.

This is even more important in the light of attempts to devise molecular tools capable of modulating the activation of this transcription factor, and certainly attempts to devise molecular tools capable of modulating the activation of this transcription factor will benefit of this information [26].

However, much more work is needed to elucidate all this.
